# Smart Sticker Ultra-Low-Power Shock Detection in the Supply Chain

**DOI:** 10.3390/s22114003

**Published:** 2022-05-25

**Authors:** Tomislav Matić, Josip Zidar, Ivan Aleksi, Drago Žagar

**Affiliations:** Faculty of Electrical Engineering, Computer Science and Information Technology Osijek, Josip Juraj Strossmayer University of Osijek, Kneza Trpimira 2B, HR-31000 Osijek, Croatia; tmatic1@ferit.hr (T.M.); ivan.aleksi@ferit.hr (I.A.); drago.zagar@ferit.hr (D.Ž.)

**Keywords:** shock detection, low-power, embedded system, package, supply chain, real-time monitoring

## Abstract

This paper presents a shock detection device for packages in the supply chain. The primary purpose is to identify package damage during storage, delivery, and handling. Additionally, products are likely to be damaged if dropped from a certain height, which sometimes does not appear on the package. By continuously measuring package vibrations and detecting shocks in the supply chain, consumers can gain an insight into the state of the product upon delivery. This paper presents the Smart Sticker implementation for ultra-low-power shock detection in the supply chain. The overall energy consumption must be kept as low as possible while continuously sensing the presence of shock to ensure that the Smart Sticker’s battery lasts as long as possible. The Smart Sticker functions in three modes to meet the established constraints: low-power, active, and data transfer mode. While detecting the shock, the low-power mode uses the least amount of energy needed. If the shock exceeds the threshold, the Smart Sticker enters active mode, stores the detected g force value in memory, and then switches back to low-power mode. Finally, employing Near Field Communication (NFC) and energy harvesting, the data transfer mode allows the consumer to read the recorded data. The results show that the Smart Sticker for shock detection performs according to set requirements and successfully monitors and detects shock for packages in the supply chain.

## 1. Introduction

The supply chain is a vast and complex process that consists of several stages. The main stages include transportation, warehousing, inventory, packaging, information, and control. Packaged products in the supply chain bear a particular risk of physical damage owing to different hazards that include shocks, vibrations, temperature, humidity, light, and pressure [1,2,3].

An embedded system must measure, process, and record the necessary data to monitor the possibility of hazard events. Recorded data must be available to the consumer on-demand. Depending on product type in the supply chain, different hazardous events are important to the consumer for monitoring. Usually, embedded systems are applied to solve the proposed tasks. Subsequently, the embedded system must satisfy different constraints: the long lifespan of the device battery, energy harvesting, small physical size, price, the size of memory for data storage, and the security and integrity of stored data.

Authors in [4,5,6] present a Smart Sticker device for product environmental condition monitoring. The proposed device uses various sensors for the measurement of different physical properties. An instance of a Smart Sticker device depends on the application, i.e., the type and the number of sensors vary. For instance, when a package of frozen fish is monitored in the supply chain, the Smart Sticker device has one or more temperature sensors, with the battery resistant to low temperatures [2,3,6].

Due to ever-increasing economic trading and the increasing volume in supply and demand of products, transportation is conducted more promptly while using the stacking method for storage and transportation. Handling large pallets of stacked product boxes leads to more frequent product damage caused by vibrations (imbalance in the load, irregular surfaces) and shocks (falls, drops) during handling and transportation [7]. To investigate this problem, authors in [8,9,10] use different simulation techniques to analyze possible vibrations and shock damage of a particular product. Additionally, several standards are defined for packaging materials to prevent or reduce the damage caused by different hazards. Standards describe the procedure and apparatus for testing the packaging material. The most commonly employed standards are those by the American Society for Testing and Materials, e.g., [11], and the International Organization for Standardization, e.g., [12,13]. Furthermore, the effects of vibrations and shock are related to energy that can cause product damage. The amount of energy depends on several factors that include acceleration, frequency, position, stack height, etc. [14].

This paper proposes a Smart Sticker device for real-time shock detection. The device is based on a low-power embedded system design and operates in three modes: low-power, active, and data transfer mode. It satisfies all set requirements: the long lifespan of the device battery, energy harvesting, small physical size, price and the amount of memory for data storage. Because of its small dimensions, the proposed device can be applied to the shipped product with adhesive tape. However, the double-sided tape may not be sufficient for longer journeys in some chemically hazardous environments with the presence of solvent-containing vapors. In such cases, different adhesive materials will be used. The device is tested in laboratory conditions and on the package shipped with a local shipping company for a distance of about 1600 km back and forth.

The rest of this paper is organized as follows. Section 2 provides a literature overview of the paper topic. The proposed Smart Sticker device for shock and vibration detection is outlined in Section 3. The setup, experimental results, and the discussion are given in Section 4. Finally, Section 5 concludes the paper by stating the drawn conclusions and providing remarks for future work.

## 2. Related Work

Several authors have proposed devices for environmental condition monitoring. In [15], authors present an IoT system based on RFID technology with temperature and humidity sensors. The system is utilized to trace perishable food in the supply chain. The proposed device is based on the Raspberry Pi system. However, it does not meet low-power requirements and has small dimensions. Authors in [16] proposed an RFID tag for its “info tracing system”. The RFID tags are used to identify the cheese on which quality analyses are performed. No real-time information about the environmental conditions of the product is processed with the proposed device. Consumers can obtain information about the cheese quality from a developed web-based system. Authors in [17] presented an embedded system based on the MSP430F microcontroller from Texas Instruments, where an RFID 13.56 MHz transponder and several sensors are used for food traceability and cold chain monitoring purposes. A complex programmable logic device is used for low-power communication and a 25 mAh battery as the power source. The maximum lifetime of the proposed real-time device was less than 35 h with only temperature measurements every 2 min on the Frankfurt to Vitoria trip. Authors in [18] implemented food quality monitoring by tracing product freshness using temperature and humidity sensors. Additionally, a wireless sensor network is used to track the location of a product. Another RFID-based approach is presented in [19]. The authors developed an irreversible critical temperature indicator with an RFID transponder for wireless data transfer. Similarly, a single-use chemical shock detector was developed by WAN-YO, Asia’s leading shock indicator manufacturer. This chemical shock detector activates by turning red irreversibly when the shock intensity exceeds the g force threshold. The offered shock g-force thresholds are 5, 37, 50, 75, and 100 g. While the suggested gadget is inexpensive, it does not track the impact’s timestamp. In [20], the DB Schenker Smartbox commercial service is presented. It is a proprietary real-time system used for DB Schenker freight services to track and monitor cargo conditions. The hardware specifications are not given, and it is unavailable for use outside the DB Schenker system. In [14], authors provide a review on the topic of stacked fruit in transit that is damaged by vibrations. The authors intended to reduce fruit quality deterioration caused by vibrations in the range of 0 to 10 Hz. Additionally, the authors proposed using simulation approaches to minimize the possible damage. In [21], the authors examined using alternative ecologically-friendly materials while lowering packing costs to protect objects from shock during transit and handling. The goal was to lower the amount of material utilized while enhancing the strength of the material. The shock sensor is coupled to a weight dropped on the tested package. A force of 1 g was applied to 10 different packaging materials.

In recent years, different simulation techniques have been used to study vibration and shock effects on products in the supply chain. The first type uses special hardware that emulates vibrations and shocks in a laboratory environment. A vibration table or a shaker is used for exerting vibration effects [9,22,23]. The second approach employs computers and simulation software to analyze possible damages to certain products. In [24], authors used finite element software ABAQUS to simulate a model of shock applied to home appliances. With the executed simulations, the performance of the package liner is tested. Authors in [25] used finite element analysis to verify the model behavior of a 9 m free drop and puncture conditions set on casks used for the transport of radioactive materials. The analysis is performed using two different simulators, ABAQUS and LS-DYNA3D (presently ANSYS). Results from the two simulators are compared to verify the model’s behavior.

Various authors have measured vibration levels in transport. In [26], authors measured vibrations caused by trucks on the road as a function of truck speed, road conditions, and load levels. Peaks of the vibrations are detected between 3 and 3.5 Hz. Similarly, authors in [27] measured the vibration inside three different kinds of trucks. Their goal is to model Korea’s truck transport environment using statistical analysis and a power spectral density profile. Acceleration data are acquired using a commercial acceleration recorder [28] held with adhesive tape. The developed model profile gives similar results compared with the results presented by international standards. Authors in [29] proposed a supervised machine learning technique for shock detection to classify road roughness under ISO 8608:2016. The authors used high-performance laser scanners with a precision of <1 mm in this research. The shock detection strategy is applied to two-wheeled vehicles. The proposed method can detect a special vehicle event on the road based on road roughness, vehicle velocity, and spring deflection.

Authors in [30] used a linear array of three piezoelectric sensors to detect the shock in composite plates. The proposed method uses deep learning to determine the shock’s energy and location.

It is necessary to define the amount of mechanical stress that a product in a supply chain can tolerate when detecting shock. This is usually expressed in units of g, which represents multiples of 9.81 ${m/s}^{2}$ acceleration. The maximum value of shock expressed in g-forces for a certain product category is stated in [8,31,32] with the term fragility. However, among the three proposed sources, the categories are slightly different. Table 1 depicts values for several categories. Vibrations are usually caused by product transportation. Peak values of g’s caused by vibrations are under 1 g. Additionally, the frequencies of the peak values of vibrations and shock are under 100 Hz [14,26,27].

The main contributions of this paper are as follows. A design is proposed for a real-time device that enables acceleration monitoring and detection of shock on packages in the supply chain during storage, delivery, and handling. Furthermore, because battery power operation is necessary, Smart Sticker is designed in accordance with ultra-low-power constraints that enable longer operation times. Moreover, the developed device is tested in a laboratory environment with several test types. Finally, Smart Sticker for shock detection is tested in a real shipping environment where it is performed according to specifications.

## 3. Smart Sticker for Shock Detection

Smart Sticker is an ultra-low-power device designed to monitor several environmental conditions. It is a standalone device powered by Li-ion battery technology that stores the sensor data in internal memory and transfers it to the user via RFID communication. Depending on the installed sensors, it can measure and record the following data in real-time: temperature, humidity, pressure, light intensity, vibration, and shock. The Smart Sticker device should adhere to the package or product before shipping. After the Smart Sticker device arrives at its destination, the user can read and check the data for possible hazards.

**Figure 1:**
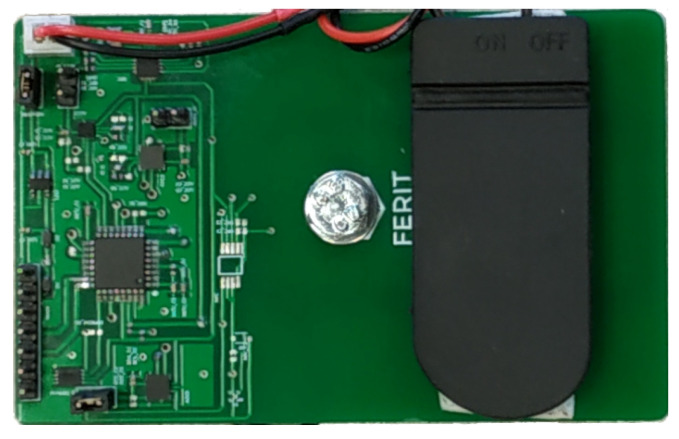
The Smart Sticker prototype.

The Smart Sticker prototype depicted in Figure 1 is about the same size as a standard credit card. The thickness is 1 cm, while the width and height are 5.4 and 9.7 cm, respectively. The Smart Sticker weighs 26.5 g when combined with two CR2032 90 mAh batteries. For different instances of the Smart Sticker design, however, multiple sizes and weights are feasible. The Smart Sticker’s size is determined by the number and type of sensors, while the battery type and capacity of the device determine the battery’s size and weight. Additionally, a flexible PCB design and smaller NFC antenna can be used to decrease the overall dimensions and weight. For the final design of the Smart Sticker, flexible housing will be designed and built using a stereolithography resin 3D printer. This will provide additional protection and secure the Smart Sticker components. Current prototype components are protected with a plastic spray which is sufficient for tests in the presented research.

**Figure 2:**
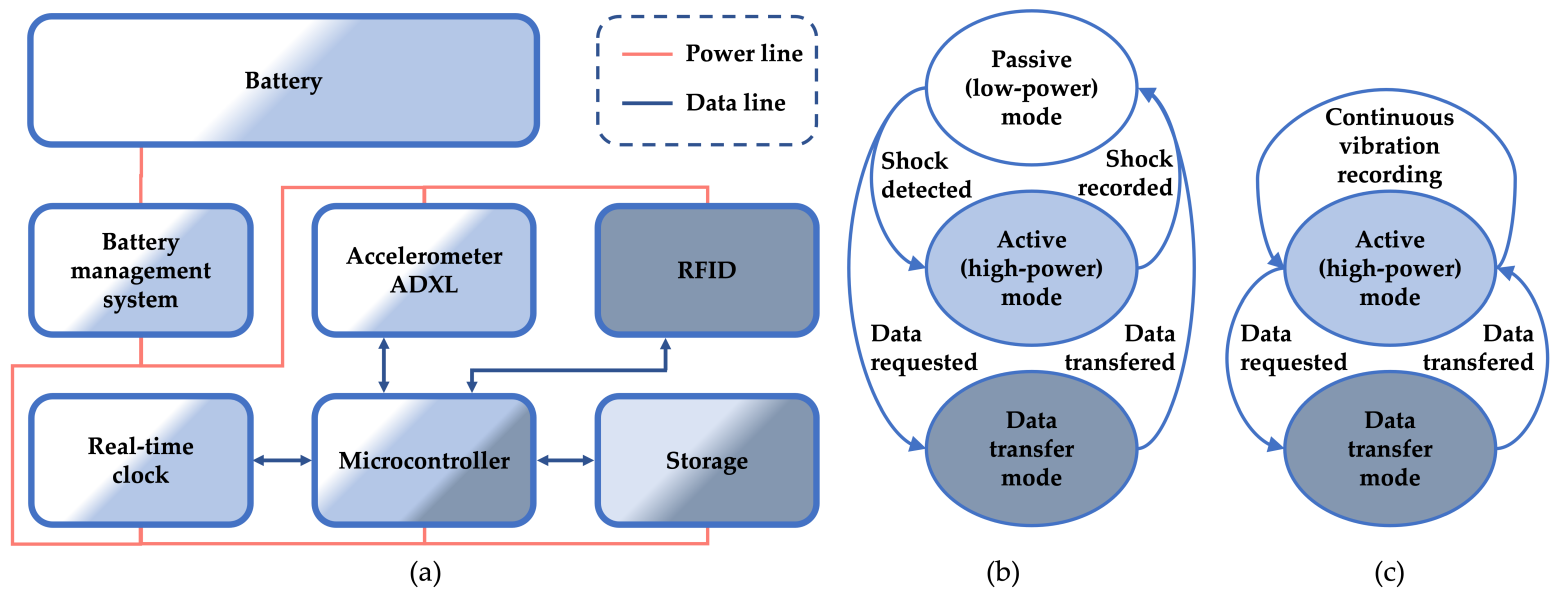
Smart Sticker device block diagram (**a**) used for: shock detection (**b**); continuous vibration recording (**c**).

The Smart Sticker for shock detection consists of several parts depicted in Figure 2. For acceleration measurement, the ADXL372 sensor is used. It has several low-power modes, scale of ±200 g that satisfies g values given in Table 1, sampling rate ranging from 400 Hz to 6400 Hz and a 12 bit data output [33]. The low-power real-time clock is used for calendar time measurement, ARM-based low-power microcontroller processes data, while low speed I2C is used for communication between components.

Firstly, when the Smart Sticker device is in the *passive mode* of operation, the ADXL372 measures acceleration continuously. It uses the low-power FIFO mode with the ability to wake up the microcontroller after measured acceleration exceeds a predefined threshold level. The wake-up event sets the Smart Sticker in the *active mode* of operation. The microcontroller analyzes the acceleration data and records the current time and acceleration values in EEPROM storage, after which it enters a low-power mode of operation.

Secondly, when the Smart Sticker device is in the *active mode* of operation, it can measure acceleration, i.e., vibration data continuously. After the measured g force exceeds a predefined threshold level, the shock acceleration value and time of the shock event are stored in EEPROM memory. Because all device components, except the RFID, are in an active mode of operation, it consumes a significant amount of energy.

Finally, when the Smart Sticker device is in the *data transfer mode* of operation, users can read the data using wireless RFID or wired UART communication protocols. When an RFID device is present, the battery management system detects the RF field from the device, disables battery power, and enables power supply from the RFID reader IC. Energy harvested from the RF field is sufficient for the data transfer operation. With the absence of the RF field, the battery management system switches power supply back to the battery. Therefore, the energy is harvested from the device reading the data (e.g., mobile phone with NFC feature), and the battery energy is conserved. Similarly, for the wired UART communication, the battery management system detects the power from the UART reading device and uses the reading device’s power supply for the data transfer.

**Figure 3:**
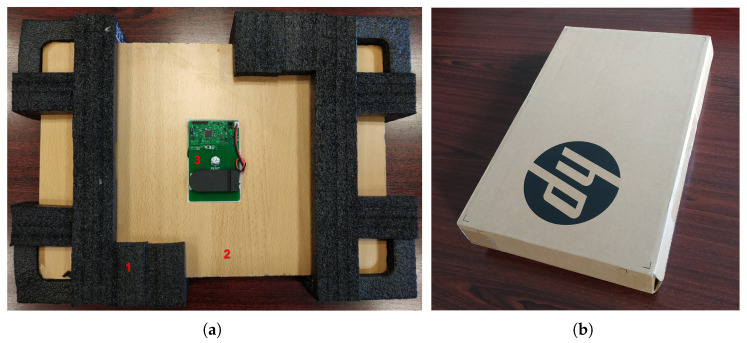
Smart Sticker prototype for shock detection on figure (**a**) (marked with 3) mounted on a testing package where 1 marks the cushioning material and 2 marks the ply board. Original cardboard box on figure (**b**).

A rigid PCB prototype is developed and used for testing in a laboratory environment. Figure 3a shows the Smart Sticker prototype for shock detection mounted on a testing package. The plywood in Figure 3a has the same weight and dimensions as a $15^{\prime \prime }$ laptop. The cushioning material of the package is an original packaging material of an HP $15^{\prime \prime }$ laptop. For testing purposes, plywood and the cushioning material are inserted in an original cardboard box, cf. Figure 3b. The Smart Sticker prototype can be mounted on the plywood, cf. Figure 3a, or on the cardboard box of the package, cf. Figure 3b. The price of the Smart Sticker instance with the ADXL372 acceleration sensor used in this research is approximately EUR 30. For extremely fragile products with a set fragility level of 15 g, a less expensive acceleration sensor can be used with an approximate Smart Sticker price of EUR 20. Considering the prices, it makes sense to use the device for more expensive single products in the supply chain, e.g., flat-screen TV, or for a pallet of less expensive products. The given prices are based on 10 produced samples. For larger production quantities, a price reduction is possible.

Acceleration values are measured for x(t),y(t),z(t) axes and M(t) value for all axes is calculated with

(1) $$M\left(t\right)=\sqrt{x{\left(t\right)}^{2}+y{\left(t\right)}^{2}+z{\left(t\right)}^{2}}.$$

**Figure 4:**
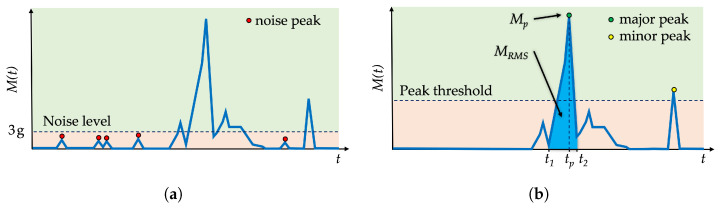
Example of acceleration magnitude values M(t): before noise canceling (**a**); peak detection after noise canceling (**b**).

The acceleration sensor has a measurement inaccuracy of ±3 g. This measurement noise is visible in the acceleration’s magnitude M(t) graph when measuring the acceleration of 0 g, cf. Figure 4a. Firstly, a noise-canceling technique sets all noise peaks to zero, thus removing the unwanted noise. Secondly, the peak detection technique is applied to identify all peaks above the peak threshold. Maximum peak value $M_{p}$ is considered as the major peak with the highest *g* value, i.e., the highest shock value applied to the package, cf. Figure 4b. Major peak value $M_{p}$ indicates a possible shock hazard at the time instance $t_{p}$ for a specific product category according to the list of products in Table 1.

Another value that is calculated from the acceleration magnitude graph M(t) is the root mean square $M_{RMS}$ of the major peak, cf. blue area on Figure 4b. The $M_{RMS}$ value provides additional insight into the possible damage to the package. For example, if the package dropped two times, with both impacts having similar peak values, then the comparison of $M_{RMS}$ values decides which peak is more significant for recording in the memory.

For the example given in Figure 4b, the detected shock has the major peak occurring at the time instance $t_{p}$. The corresponding major peak value $M_{p}$ is defined by

(2) $$M_{p}=\max_{t\in [t_{1},t_{2}]}M\left(t\right).$$

The root mean square (RMS) of the detected major peak is calculated with

(3) $$M_{RMS}=\sqrt{\frac{1}{n}\sum_{t=t_{1}}^{t_{2}}M\left(t\right)},$$

(4) $$n=\frac{t_{2}-t_{1}}{Ts}+1,$$

where $t_{1}$ and $t_{2}$ are the time instances of the major peak’s start and end, *n* is the number of samples within time interval $\left[t_{1},t_{2}\right]$, and $T_{S}$ is the sampling period. Finally, the detected shock with the corresponding major peak values: $M_{RMS}$, $M_{p}$, and $t_{p}$, are stored in the Smart Sticker’s memory.

## 4. Experimental Results

**Figure 5:**
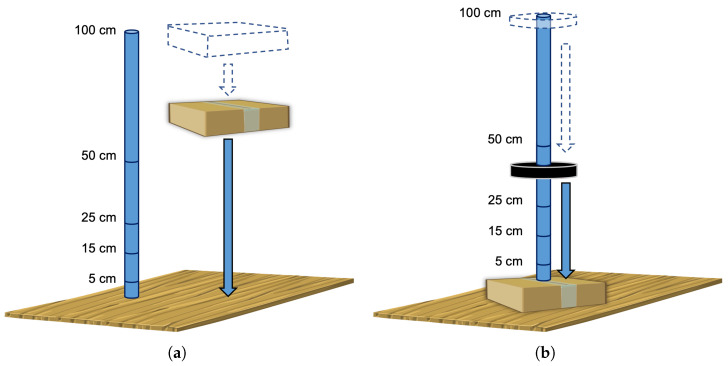
Shock detection test in a laboratory environment: package drop to the floor (**a**); weight drop on the package (**b**).

Several experiments are conducted to determine the Smart Sticker’s ability to measure shock. Tests are carried out in a laboratory environment using the package shown in Figure 3b. The measuring tool is used to drop the package from different heights on a flat hardwood floor, cf. Figure 5. The package is dropped horizontally and vertically to simulate dropping of the package during transportation and storage, cf. Figure 5a. Additionally, a measuring tool is used to drop a round weight on the package placed horizontally on the hardwood floor, cf. Figure 5b. This simulates the possible impact of the package by another package. Finally, one test is carried out in a real shipping environment. A package with the Smart Sticker is sent on a journey from Osijek, Croatia, to Dubrovnik, Croatia, and back. The length of the one-way trip is approximately 800 km and the duration about 5 days.

**Figure 6:**
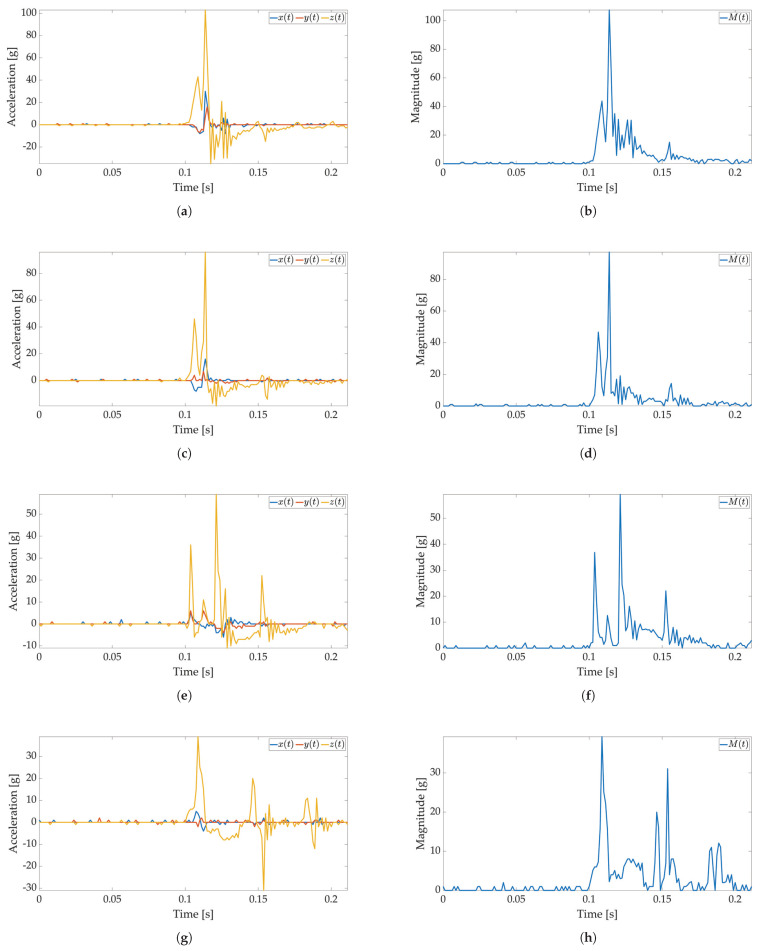
Shock detection values for package falling horizontally from various heights (800 Hz in box without FIFO). Acceleration axes on figures (**a**) (100 cm), (**c**) (50 cm), (**e**) (20 cm), (**g**) (10 cm) and acceleration magnitude waveform on figures (**b**) (100 cm), (**d**) (50 cm), (**f**) (20 cm), (**h**) (10 cm).

**Figure 7:**
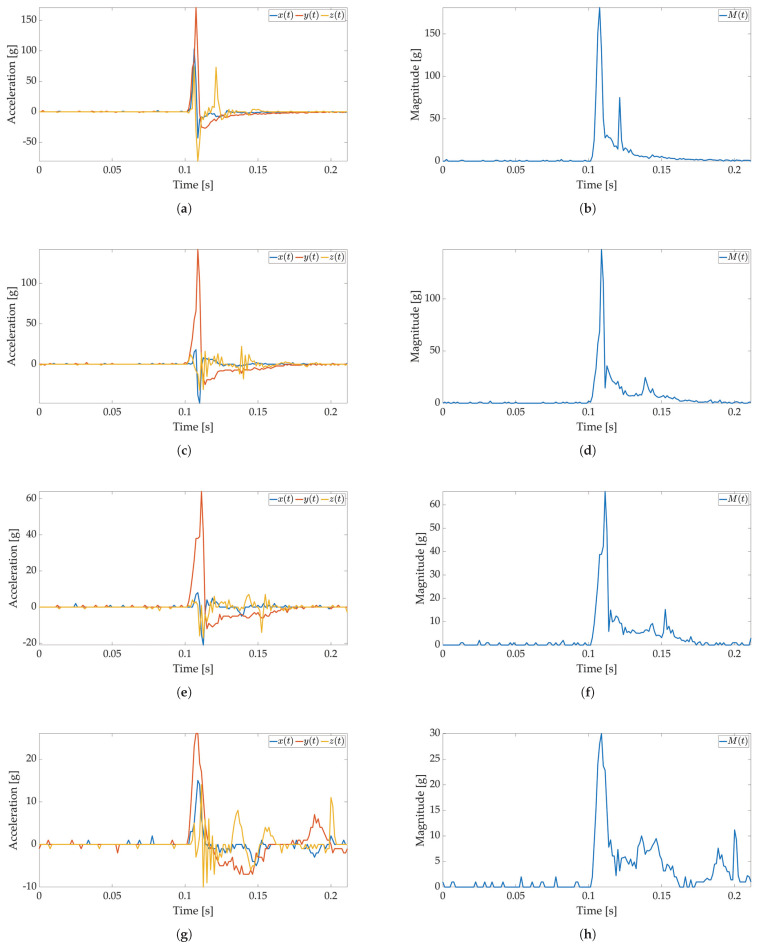
Shock detection values for package falling vertically from various heights (800 Hz in box without FIFO). Acceleration axes on figures (**a**) (100 cm), (**c**) (50 cm), (**e**) (20 cm), (**g**) (10 cm) and acceleration magnitude waveform on figures (**b**) (100 cm), (**d**) (50 cm), (**f**) (20 cm), (**h**) (10 cm).

In the first test, only active mode is used, and no power savings are applied. The maximum available sampling frequency is set for the ADXL sensor when used with I2C communication (800 Hz). The major threshold value is set to 5 g, and 170 values are saved to Smart Sticker storage. The Smart Sticker is placed inside the package and adhered to the plyboard. The package is dropped vertically and horizontally from different heights. Every drop is repeated 4 times. This test is conducted to show the connection between x(t),y(t),z(t) and M(t) values, to evaluate the time interval of the major peak and to find the necessary number of samples for shock evaluation. Selected results for *X*, *Y*, *Z* axes and RMS values for tested heights are depicted in Figure 6 and Figure 7. The highest acceleration values are visible in *Z*-axes for horizontal (*Z*-axis is oriented up/down) and in *Y*-axes for vertical (*Y*-axis is oriented up/down) drop as expected, while the measured acceleration for other axes is much lower. For a horizontal drop height of 50 cm and higher, the g values are above 80 g, resulting in possible damage to the packaged device, cf. Table 1. Additionally, for the vertical drop, a smaller drop height of 20 cm results in acceleration values greater than 60 g. The reason for this can be found in the shape of the used package shown in Figure 3b. For vertical drop, the impact surface is much smaller than the impact surface of the horizontal drop; therefore, more force is transferred to the package in the vertical drop test. Additionally, a package can hit the floor at an angle, resulting in a much lower impact surface and higher acceleration values. Based on Equation (1) for all *X*-, *Y*-, *Z*-axes measurements M(t) values are calculated and shown in Figure 6b,d,f,h for horizontal and Figure 7b,d,f,h for vertical drop. The major peak $M_{p}$ value is approximately the same when compared with the original *X*-, *Y*-, *Z*-axes measurements. Therefore, only M(t) values are considered for all other tests in this paper. Moreover, the shock duration for all drop heights is approximately the same (≈50 ms), while the duration of the major peak is much less than the total measurement time (≈8 ms). The number of samples that can record at least 50 ms of acceleration values for the selected sampling frequency is sufficient to detect the major peak value.

**Figure 8:**
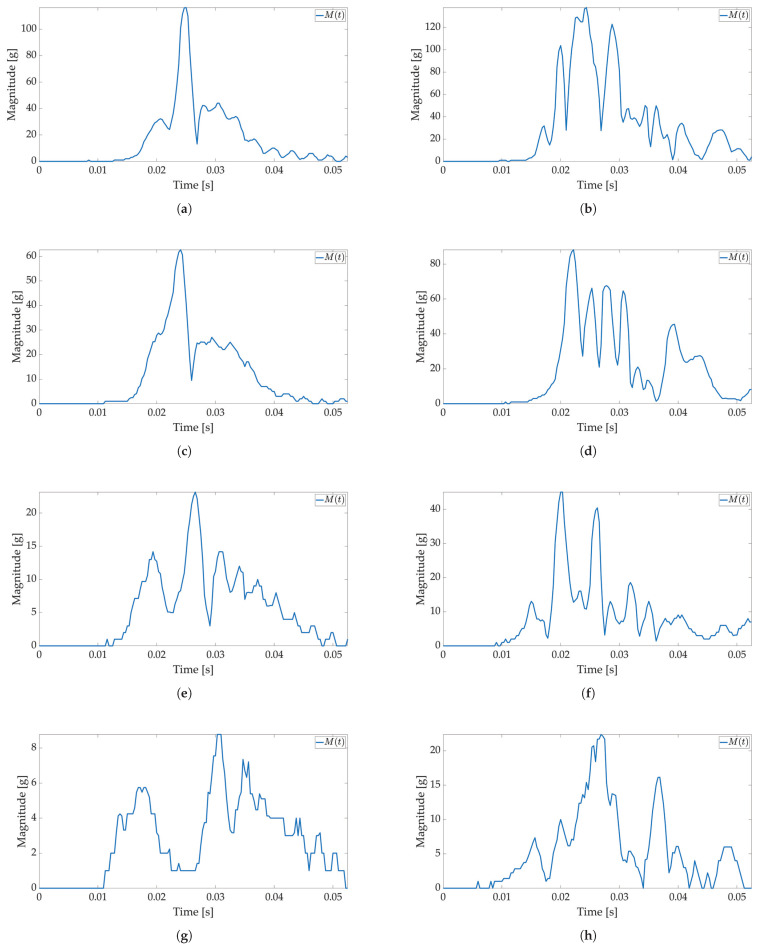
Shock detection values for package falling horizontally from various heights (3200 Hz with FIFO). Acceleration magnitude waveform: measured inside the package on figures (**a**) (100 cm), (**c**) (50 cm), (**e**) (20 cm), (**g**) (10 cm); measured on the package on figures (**b**) (100 cm), (**d**) (50 cm), (**f**) (20 cm), (**h**) (10 cm).

**Figure 9:**
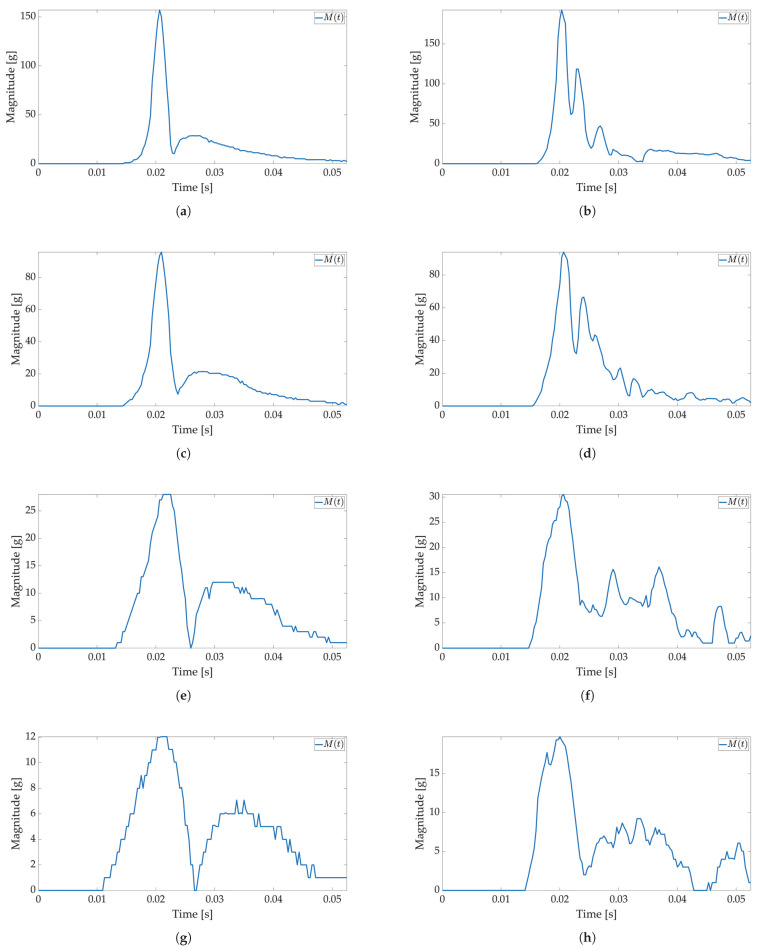
Shock detection values for package falling vertically from various heights (3200 Hz with FIFO). Acceleration magnitude waveform: measured inside the package on figures (**a**) (100 cm), (**c**) (50 cm), (**e**) (20 cm), (**g**) (10 cm); measured on the package on figures (**b**) (100 cm), (**d**) (50 cm), (**f**) (20 cm), (**h**) (10 cm).

**Figure 10:**
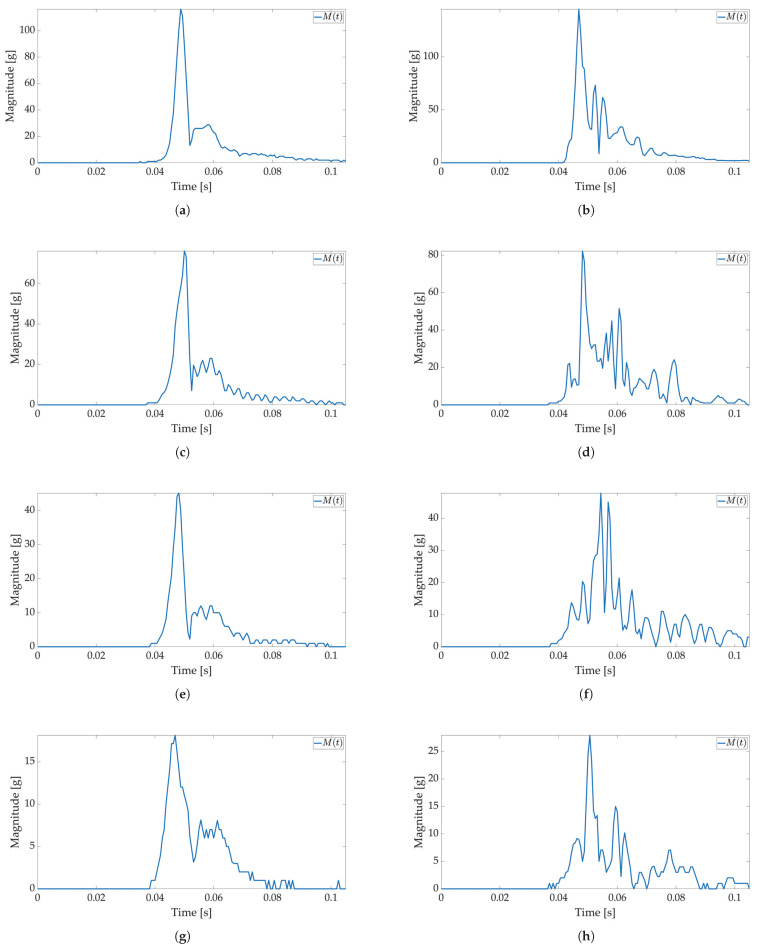
Shock detection values for package falling horizontally from various heights (1600 Hz with FIFO). Acceleration magnitude waveform: measured inside the package on figures (**a**) (100 cm), (**c**) (50 cm), (**e**) (20 cm), (**g**) (10 cm); measured on the package on figures (**b**) (100 cm), (**d**) (50 cm), (**f**) (20 cm), (**h**) (10 cm).

**Figure 11:**
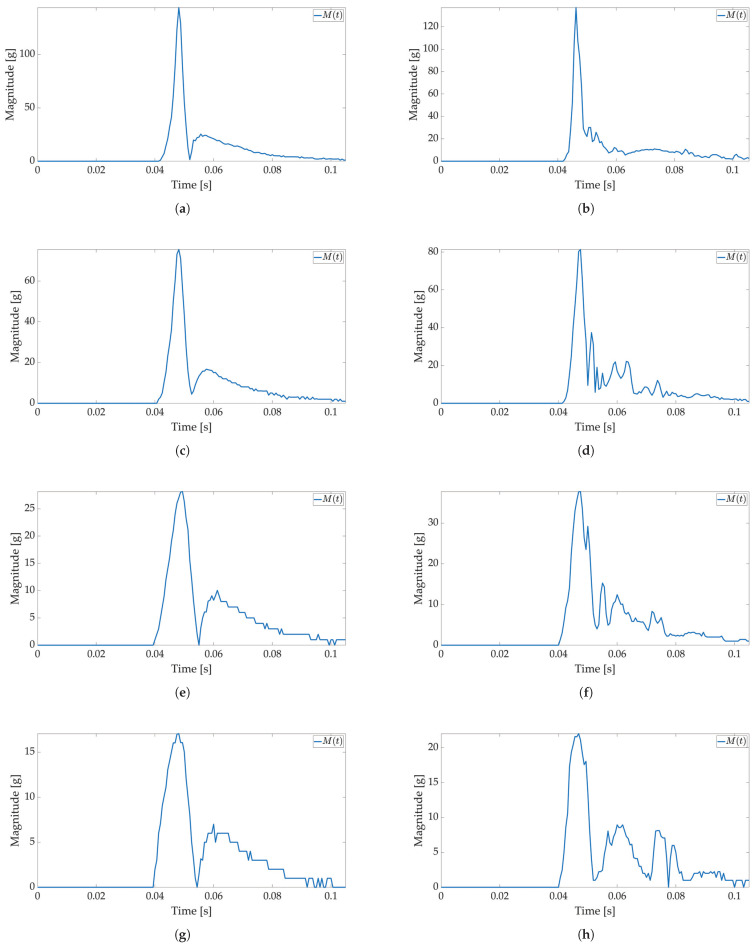
Shock detection values for package falling vertically from various heights (1600 Hz with FIFO). Acceleration magnitude waveform: measured inside the package on figures (**a**) (100 cm), (**c**) (50 cm), (**e**) (20 cm), (**g**) (10 cm); measured on the package on figures (**b**) (100 cm), (**d**) (50 cm), (**f**) (20 cm), (**h**) (10 cm).

**Figure 12:**
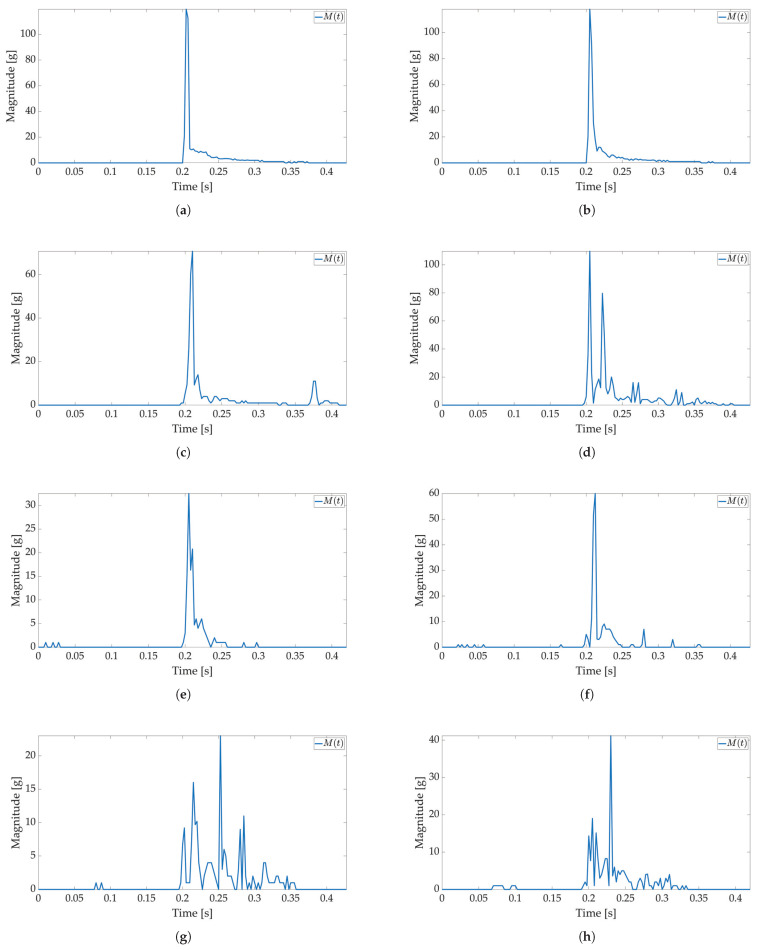
Shock detection values for package falling horizontally from various heights (400 Hz with FIFO). Acceleration magnitude waveform: measured inside the package on figures (**a**) (100 cm), (**c**) (50 cm), (**e**) (20 cm), (**g**) (10 cm); measured on the package on figures (**b**) (100 cm), (**d**) (50 cm), (**f**) (20 cm), (**h**) (10 cm).

**Figure 13:**
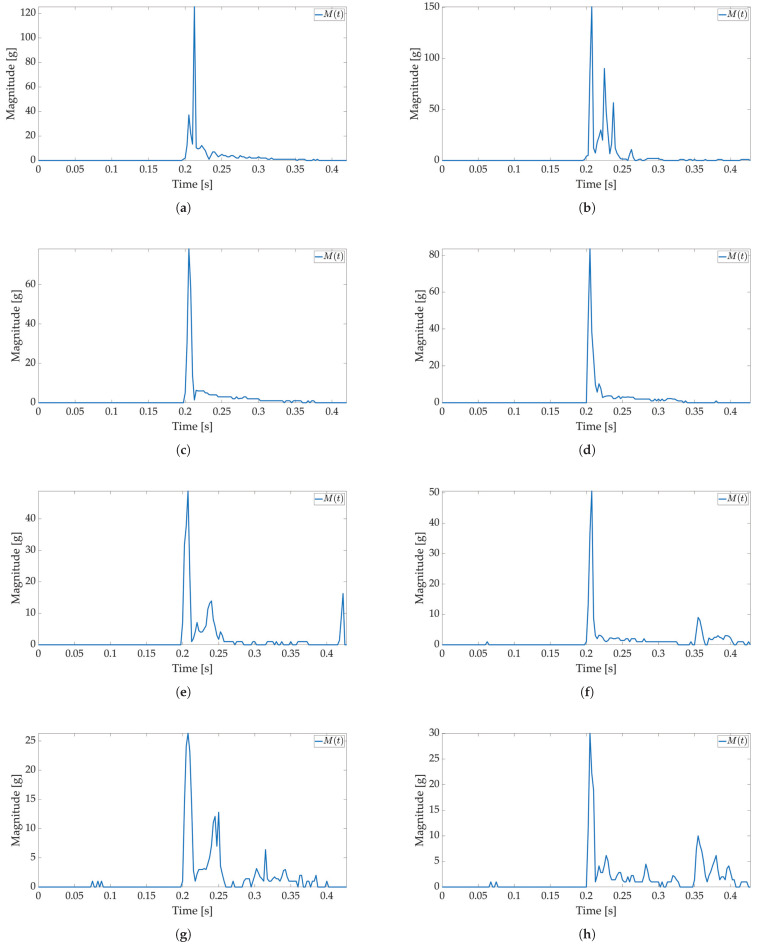
Shock detection values for package falling vertically from various heights (400 Hz with FIFO). Acceleration magnitude waveform: measured inside the package on figures (**a**) (100 cm), (**c**) (50 cm), (**e**) (20 cm), (**g**) (10 cm); measured on the package on figures (**b**) (100 cm), (**d**) (50 cm), (**f**) (20 cm), (**h**) (10 cm).

In the second test, low-power and active mode are used to enable Smart Sticker power saving. In the low-power mode acceleration sensor, the real-time clock and battery management system are active, the microcontroller is in a power-saving mode, while storage and RFID are off, cf. Figure 2. The FIFO feature of the ADXL sensor is used for power saving, while interruptions on the set major threshold sets the Smart Sticker in the active mode to record the shock data. The FIFO feature of the sensor enables the use of higher sampling frequencies and it is not limited by the I2C communication protocol, while the FIFO buffer limits the maximum number of saved acceleration values for each axis to 170 samples. The results of the first test show that the duration of the shock is ≈50 ms; therefore, the maximum sampling frequency in FIFO mode is ≈170/50 ms = 3400 Hz. The closest ADXL sensor sampling frequency of 3200 Hz is used. Moreover, the duration of the major peak is ≈8 ms when converted to frequency ≈125 Hz. According to the Nyquist–Shannon theorem, the signal sampling frequency must be at least twice the maximum signal frequency [34]. In practice, for non-periodic signals, the signal sampling frequency is several times greater. Therefore, sampling frequencies of 1600 Hz (12.8 times greater) and 400 Hz (3.2 times greater) are used with the same number of saved samples to analyze the possible differences in the major peak value as sampling frequency decreases. The Smart Sticker is placed inside the package (adhered to the plyboard) and outside the package (adhered to the box). Measurements for the highest sampling frequency (Figure 8 and Figure 9) show M(t) graph of the shock in most detail. One major peak is always visible in M(t) measurements, and the highest measured magnitude value is above 150 g (cf. Figure 9b), which corresponds to the values presented in the first test. A lower acceleration sampling frequency results in lower energy consumption for the Smart Sticker low-power mode. Therefore, 1600 Hz (Figure 10 and Figure 11) and 400 Hz (Figure 12 and Figure 13) sampling frequencies are tested. There is no significant difference in the major peak value of the M(t) graph when compared with the 800 Hz frequency for the first test (Figure 6 and Figure 7) or the 3200 Hz frequency in the second test. For 400 Hz M(t) graph (Figure 12 and Figure 13) does not show smaller peaks visible in M(t) graphs for frequencies greater than 800 Hz. Therefore, the minimum sampling frequency for shock detection is 800 Hz. Differences in the M(t) values for tests carried out with the Smart Sticker placed inside and outside the package are due to the cushioning material protecting the goods. From the results in Figure 10, Figure 11, Figure 12 and Figure 13 it can be seen that the M(t) values are similar, but in most cases, $M_{p}$ values are greater when measured outside the package as expected.

**Figure 14:**
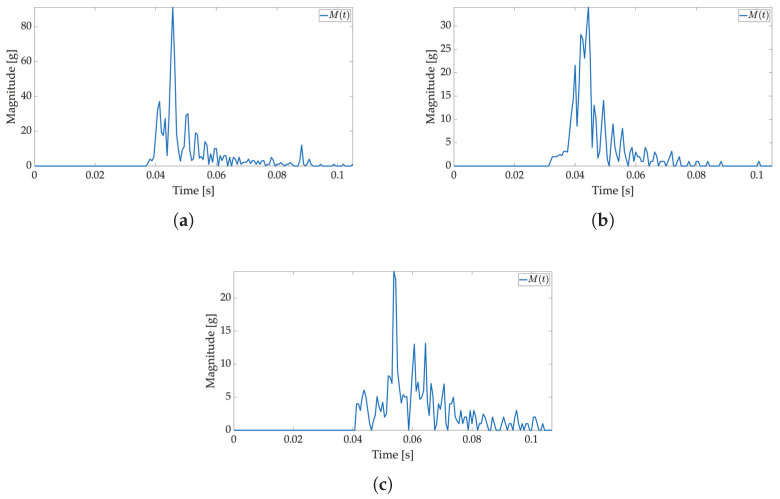
Shock detection values for weight drop onto the stationery package (1600 Hz with FIFO). The weight falls from the different heights listed below. Acceleration magnitude waveform measured inside the package on figures (**a**) (100 cm), (**b**) (50 cm), (**c**) (20 cm).

In the third test, a weight is dropped on the package to simulate the impact of a moving object on the package with the Smart Sticker, cf. Figure 5b. This is a possible scenario during transport. Another object (package) in the container or a truck wagon can fall on the shipped package and possibly damage the content. The device used in this test is shown in Figure 5b. The weight used has a mass of 1.25 kg and a diameter of 125 mm with a hole in the center. The weight slides from a selected height and impacts the package placed horizontally on a hardwood floor. Results are shown in Figure 14. A measurement for the weight falling from a height of 10 cm is not present because the threshold value is not exceeded when the shock occurs. The cause for this is the center of the package, which is the area of highest shock absorption. Additionally, the major peak values are somewhat smaller in intensity when compared to the package drop test for the same reason.

**Figure 15:**
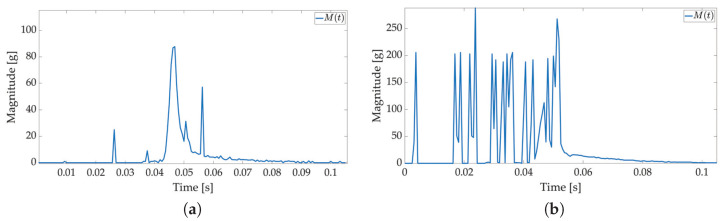
Measurements on journey from Osijek to Dubrovnik (1600 Hz with FIFO). Acceleration magnitude waveform measured inside the package on figures (**a**) (first measurement), (**b**) (second measurement).

In the fourth test, the package with a Smart Sticker prototype adhered to the product is sent via a local shipping company from Osijek, Croatia to Dubrovnik, Croatia. Two shocks are recorded in the storage. The first measurement is shown in Figure 15a where a medium-intensity shock has occurred. It corresponds to a free fall of the package from an approximate height of 50 cm when tested in the laboratory. Such shock levels could have damaging effects on some types of products listed in Table 1. The second measurement is shown in Figure 15b that does not correspond to the laboratory tests. Several shocks occurred over 150 g’s in a small period of time. One of the possible reasons is that several other objects in the container fell on the package with the Smart Sticker inside. Such a combination of shock levels can damage almost all product types if proper packing materials are not applied, cf. Table 1.

All test data saved in the Smart Sticker storage are transferred to the user for analysis using UART or RFID communication.

The Smart Sticker must be an ultra-low-power device to enable long operation during shipping. This is achieved with a specific hardware design using the ultra-low-power RTC and the ADXL sensor in low-power mode. Differences in current consumption for the Smart Sticker low-power mode and active mode are given in Table 2. Smart Sticker is in low-power mode most of the time, waiting for the set acceleration threshold value, after which it enters active mode for a fraction of the time, thus significantly saving total energy consumption. Smart Sticker current is measured with a high precision Keysight 34465A multi-meter as the average of 100 measurements. From measurements shown in Table 2, current consumption is in the worst case (for the highest acceleration sampling frequency 3200 Hz) 47 times, and in the best case (for the lowest sampling frequency 400 Hz) 91 times less in the low-power mode when compared to the active mode.

Based on the current measurement, it is possible to estimate the operation life of the Smart Sticker for shock detection. Estimation is made based on ideal conditions, without considering influences such as low or high temperature, variations in battery capacity, homogeneity of chemistry in cells, the impact of battery self-discharge, and state of health. The values given in Table 3 are based on a 90 mAh battery at 25 °C. As the acceleration sampling frequency increases, the number of estimated operation days decreases. Moreover, the number of shocks has a negligible influence on the estimated operation time. For the minimum sampling frequency of 800 Hz and an assumption of maximum 50 measured acceleration values over the set peak threshold, the estimated operation time is 194 days, sufficient for long shipping journeys. Additionally, the operation time of the Smart Sticker for shock detection can be increased, if necessary, using the higher capacity battery. For shorter local journeys, there is a possibility of reusing the device. After the user receives the package and checks the shock data, they can return the box with the adhered Smart Sticker to the shipping company. The shipping company can remove the device from the box, delete the saved data from the device and reuse it on a different package. Additionally, if rechargeable batteries are used, device operation time can be increased with charging.

## 5. Conclusions

In this paper, we present the Smart Sticker for shock detection. It is an ultra-low-power battery-powered device designed to measure and record acceleration data of a package in real-time. Acceleration data are analyzed, and shock is detected for the package in transport. The maximum magnitude peak value $M_{p}$ and RMS value of the detected $M_{p}$ are saved to the Smart Sticker memory along with the correct calendar time. Data are transferred to the user via RFID wireless or UART wired communication without additional energy consumption. Ultra-low-power is achieved with the specific hardware design in combination with the low-power mode of the acceleration sensor, and this enables a longer operational time of the Smart Sticker in the supply chain.

Several experiments are conducted that test the ability of the Smart Sticker for shock detection. The results show measurements of a falling package from different heights with different sampling frequencies. The Smart Sticker is tested on a commercial package by adherence to the product inside the package. Additionally, a test is carried out that measures shock when a weight dropped from different heights hits the package. Moreover, one package is sent via a local shipping company on a 1600 km long trip with the Smart Sticker attached inside the package.

The conducted experiments show that a minimum sampling frequency of 800 Hz is necessary for proper shock detection. Moreover, when the Smart Sticker is located inside the package, measurements show lower shock peak values than those outside the package. With the proper usage of a protective layer on the package, such as cardboard and protective foam, the package’s resilience to shocks is increased. It is noticeable that the shock of the vertical drop of the package shows higher peak values than the horizontal fall of the package. The reason for this is the significantly smaller contact area at the impact zone for the vertical drop. Additionally, weight is dropped on the package from different heights, and the measured shock results are consistent with the results of the package falling. In the test where one package is sent on a journey with the local shipping company, two high-level shock events are recorded that would result in product damage. Because the Smart Sticker must be battery-powered, current consumption is measured and the estimated operation time is given.

Although the given results show that the Smart Sticker is able to detect shock in the supply chain, several avenues for future work remain open. Firstly, it would be interesting to detect and analyze the rotation (tilt) of the package while the shock occurred. For specific packages, tilt of the package can significantly influence the damage. Furthermore, different package protections and cushioning material types should be tested and compared. For asymmetrical packages, e.g., a large L-shaped package, it is necessary to determine the proper location for the Smart Sticker. Moreover, research should be carried out on the protection and integrity of the stored shock data on the Smart Sticker data storage. Finally, several tests should be conducted in real shipping environments, including trucks, trains, airplanes, boats, etc.

## Figures and Tables

**Table 1:** Fragility expressed in g’s for different product categories.

Fragility Category	Product Type	Level
Extremely Fragile	Missile guidance systems, precision measurement instruments, large electronic computers	15–25 g’s
Very Delicate	Mechanically shock-mounted instruments, precision displays, electronic equipment	25–40 g’s
Delicate	Aircraft accessories, electric typewriters, cash registers and other electronically operated office equipment	40–60 g’s
Moderately Delicate	Television receivers, aircraft accessories, desktop computers	60–85 g’s
Moderately Rugged	Laundry equipment, refrigerators, appliances	85–115 g’s
Rugged	Machinery, ceramic ware	≥115 g’s

**Table 2:** Measured Smart Sticker current consumption with various acceleration sampling frequencies.

Sampling Frequency [Hz]	Low-Power Mode [µA]	Active Mode [mA]
400	16.869	1.540
800	19.204	1.542
1600	23.951	1.554
3200	32.897	1.565

**Table 3:** Estimated Smart Sticker operation time based on current consumption measurements in low-power and active mode for a different number of measured shocks per day.

Sampling Frequency [Hz]	Estimated Operation Time [Days]				
	5 Shocks	10 Shocks	20 Shocks	50 Shocks	100 Shocks
400	222.177	222.061	221.830	221.138	219.994
800	195.182	195.093	194.914	194.380	193.496
1600	156.508	156.450	156.334	155.989	155.416
3200	113.959	113.928	113.867	113.683	113.378

## Data Availability

Not applicable.

## Abbreviations

The following abbreviations are used in this manuscript:

NFC	Near Field Communication
RFID	Radio Frequency Identification
FIFO	First-In First-Out
UART	Universal Asynchronous Receiver-Transmitter
EEPROM	Electrically Erasable Programmable Read-Only Memory
RMS	Root Mean Square
